# Dysmorphophobia as a factor that worsens the affective state and the life quality of patients with eating disorders. The final data of the study

**DOI:** 10.1192/j.eurpsy.2021.954

**Published:** 2021-08-13

**Authors:** E. Okonishnikova, A. Bryukhin, T. Lineva, I. Belokrylov

**Affiliations:** Department Of Psychiatry And Medical Psychology, RUDN University Moscow., Moscow, Russian Federation

**Keywords:** eating disorder, body dysmorphic disorder

## Abstract

**Introduction:**

Anorexia nervosa (AN) and bulimia nervosa (BN) take one of the first places in the risk of fatal outcome among eating disorders, have a tendency to chronicity and high suicidal risk. Psychopathological basis for AN and BN is a dysmorfofobia or a pathological dissatisfaction with one’s body, characterized by intrusive, overvalued or delusional ideas of physical disability. Dysmorfofobia affects the formation of affective pathology and reduces the life quality.

**Objectives:**

The study of the correlation between the degree of dissatisfaction with one’s bodies, affective disorders and life quality of patients with AN and BN.

**Methods:**

130 female patients with AN and BN at the age of 13-44 years (the average age is 18). The disease duration from 6 months to 24 years. Validated Questionnaire image of one’s own body (QIOB) and the Scale of satisfaction with one’s body (SSOB); Hospital anxiety and depression scale (Zigmond A.); Questionnaire for the assessment of life quality (SF-36); Microsoft Excel standard correlation calculation.

**Results:**

Dissatisfaction with one’s body based on QIOB and SSOB tests has a significant positive correlation with anxiety and depression, a significant correlation with the psychological component of health, a weak correlation with the physical component of health.

**Conclusions:**

Dissatisfaction with one’s body or dysmorfofobia of patients with AN and BN significantly affects their affective state and psychological component of life quality which leads to a decrease in functioning up to social maladaptation and disability to social maladjustment. The publication was prepared with the support of the “RUDN University Program 5-100”.

